# Comparative Structures and Evolution of Vertebrate Carboxyl Ester Lipase (*CEL*) Genes and Proteins with a Major Role in Reverse Cholesterol Transport

**DOI:** 10.1155/2011/781643

**Published:** 2011-11-21

**Authors:** Roger S. Holmes, Laura A. Cox

**Affiliations:** ^1^Department of Genetics, Texas Biomedical Research Institute, San Antonio, TX 78245-0549, USA; ^2^Southwest National Primate Research Center, Texas Biomedical Research Institute, San Antonio, TX 78245-0549, USA; ^3^School of Biomolecular and Physical Sciences, Griffith University, Nathan, QLD 4111, Australia

## Abstract

Bile-salt activated carboxylic ester lipase (CEL) is a major triglyceride, cholesterol ester and vitamin ester hydrolytic enzyme contained within pancreatic and lactating mammary gland secretions. Bioinformatic methods were used to predict the amino acid sequences, secondary and tertiary structures and gene locations for *CEL* genes, and encoded proteins using data from several vertebrate genome projects. A proline-rich and O-glycosylated 11-amino acid C-terminal repeat sequence (VNTR) previously reported for human and other higher primate CEL proteins was also observed for other eutherian mammalian CEL sequences examined. In contrast, opossum CEL contained a single C-terminal copy of this sequence whereas CEL proteins from platypus, chicken, lizard, frog and several fish species lacked the VNTR sequence. Vertebrate *CEL* genes contained 11 coding exons. Evidence is presented for tandem duplicated *CEL* genes for the zebrafish genome. Vertebrate CEL protein subunits shared 53–97% sequence identities; demonstrated sequence alignments and identities for key CEL amino acid residues; and conservation of predicted secondary and tertiary structures with those previously reported for human CEL. Phylogenetic analyses demonstrated the relationships and potential evolutionary origins of the vertebrate *CEL* family of genes which were related to a nematode carboxylesterase (*CES*) gene and five mammalian *CES* gene families.

## 1. Introduction

Bile-salt activated carboxylic ester lipase (CEL; also designated as cholesterol esterase and lysophospholipase) is a major triglyceride, cholesterol ester and vitamin ester hydrolytic enzyme contained within pancreatic and lactating mammary gland secretions [5–10]. CEL is also secreted by the liver and is localized in plasma where it contributes to chylomicron assembly and secretion, the selective uptake of cholesteryl esters in HDL by the liver, LDL lipid metabolism, and reverse cholesterol transport [11–14]. Plasma CEL may also contribute to endothelial cell proliferation, the induction of vascular smooth muscle proliferation, and thrombus formation through interaction with platelet CXCR4 [15]. More recently, CEL expression has been reported in human pituitary glands where it may function in regulating hormone secretion in association with the CEL hydrolytic activity of ceramides [16]. 

Structures for several human and animal *CEL* genes and cDNA sequences have been determined, including human (*Homo sapiens*) [7, 17–19], gorilla (*Gorilla gorilla*) [20], mouse (*Mus musculus*) [21–23], rat (*Rattus norvegicus*) [24–26], and cow (*Bos taurus*) *CEL* genes [1, 27]. The human *CEL *gene comprises 11 exons and is localized on chromosome 9 [28]. Several Alu repetitive sequence elements and putative transcription factor binding sites have been identified in the 5′-untranslated (UTR) region, including pancreatic-specific binding sites, which contribute to a high level of expression in the exocrine pancreas [17, 29, 30]. Exon 11 of the human *CEL* gene encodes a variable number of tandem repeat sequences region (VNTR) (17 repeats are most common) which is highly polymorphic in human populations and contributes to plasma cholesterol and lipid composition [13]. Moreover, rare *CEL* gene defects in this region are responsible for a monogenically derived diabetes condition called maturity-onset diabetes of the young type 8 (MODY8), also known as diabetes and pancreatic exocrine dysfunction (DPED), which causes a defect in insulin secretion [31, 32]. 

Human *CEL *is expressed predominantly in the lactating mammary gland and beta cells of the exocrine pancreas, where the enzyme contributes significantly to triglyceride, cholesterol ester and vitamin ester metabolism [5–10]. CEL also promotes large chylomicron production in the intestine, and its presence in plasma supports interactions with cholesterol and oxidized lipoproteins [11] which may influence atherosclerosis progression [12]. *CEL* expression has also been reported in the human pituitary gland, and a possible role for CEL in the regulation of hormone secretion and ceramide metabolism has been described [16]. Studies of *Cel^−^*/*Cel^−^* knock out mice have shown that other enzymes besides CEL are predominantly responsible for the hydrolysis of dietary cholesteryl esters, retinyl esters, and triglycerides [33]. Metabolic studies of *Cel*-null mice however have reported that a lack of CEL activity causes an incomplete digestion of milk fat and lipid accumulation by enterocytes in the ileum of neonatal mice which suggests a major role for this enzyme in triglyceride hydrolysis in breast-fed animals [9, 34]. Moreover, reverse cholesterol transport is elevated in carboxyl ester lipase-knockout mice which supports a significant role for this enzyme in the biliary disposal of cholesterol from the body [14]. 

A *CEL*-like gene (designated as *CELL*) has also been identified on human and gorilla chromosome 9, about 10 kilobases downstream of *CEL*, which is transcribed in many tissues of the body but lacks exons 2–7 and is unlikely to be translated into protein [17, 20, 35]. The *CELL* pseudogene gene duplication apparently occurred prior to the separation of Hominidae (man, chimpanzee, gorilla, and orangutan) from Old World monkeys (macaque) with *CELL* being restricted to genomes of man and the great apes [20]. 

Three-dimensional structural analyses of human CEL have shown that the enzyme belongs to the alpha/beta hydrolase fold family with several key structural and catalytic features, including an active site catalytic triad located within the enzyme structure and partially covered by a surface loop, the carboxyl terminus region of the protein which regulates enzymatic activity by forming hydrogen bonds with the surface loop to partially shield the active site, and a loop domain which binds bile salt and frees the active site to access water-insoluble substrates [1, 10, 36–38]. In both conformations, CEL forms dimeric subunit structures with active sites facing each other. The common variant of the human CEL gene contains VNTR repeats, but there is a high degree of polymorphism in the repeated region [31, 32]. While the biological function of the polymorphic repeat region is unknown, it has been suggested that it may be important for protein stability and/or secretion of the enzyme, particularly given that this region contains many O-glycosyl bonds linking carbohydrate residues to the CEL C-terminus, including fucose, galactose, glucosamine, galactosamine, and neuraminic acid residues [39]. 

This paper reports the predicted gene structures and amino acid sequences for several vertebrate *CEL* genes and proteins, the predicted secondary and tertiary structures for vertebrate CEL protein subunits, and the structural phylogenetic and evolutionary relationships for these genes and enzymes with mammalian *CES* (carboxylesterase) gene families [40, 41]. 

## 2. Methods

### 2.1. Vertebrate *CEL* Gene and Protein Identification

BLAST (Basic Local Alignment Search Tool) studies were undertaken using web tools from the National Center for Biotechnology Information (NCBI) (http://blast.ncbi.nlm.nih.gov/Blast.cgi) [42]. Protein BLAST analyses used vertebrate CEL amino acid sequences previously described (Table 1). Nonredundant protein sequence databases for several mammalian genomes were examined using the blastp algorithm, including human (*Homo sapiens*) [43], chimp (*Pan troglodytes*) [44], orangutan (*Pongo abelii*) (http://genome.wustl.edu/); the cow (*Bos Taurus*) [45], horse (*Equus caballus*) [46], mouse (*Mus musculus*) [47], opossum (*Monodelphis domestica)* [48], platypus [49], chicken (*Gallus gallus*) [50], clawed toad (*Xenopus tropicalis*) [51], and zebrafish (*Danio rerio*) [52]. This procedure produced multiple BLAST “hits” for each of the protein databases which were individually examined and retained in FASTA format, and a record kept of the sequences for predicted mRNAs and encoded CEL-like proteins. These records were derived from annotated genomic sequences using the gene prediction method: GNOMON and predicted sequences with high similarity scores for human CEL [42]. Predicted CEL-like protein sequences were obtained in each case and subjected to analyses of predicted protein and gene structures. 

BLAT (BLAST-Like Alignment Tool) analyses were subsequently undertaken for each of the predicted vertebrate CEL amino acid sequences using the University of California Santa Cruz (UCSC) Genome Browser (http://genome.ucsc.edu/cgi-bin/hgBlat) [4] with the default settings to obtain the predicted locations for each of the vertebrate *CEL* genes, including predicted exon boundary locations and gene sizes. Structures for human, mouse, and rat *CEL* isoforms (splice variants) were obtained using the AceView website (http://www.ncbi.nlm.nih.gov/IEB/Research/Acembly/index.html?human) to examine predicted gene and protein structures [2]. Alignments of vertebrate CEL sequences with human carboxylesterase (CES) protein sequences were assembled using the ClustalW2 multiple sequence alignment program [53] (http://www.ebi.ac.uk/Tools/clustalw2/index.html). 

### 2.2. Predicted Structures and Properties of Vertebrate CEL Protein Subunits 

Predicted secondary and tertiary structures for vertebrate CEL-like subunits were obtained using the PSIPRED v2.5 web site tools (http://bioinf.cs.ucl.ac.uk/psipred/) [54] and the SWISS MODEL web tools (http://swissmodel.expasy.org/), respectively [55, 56]. The reported tertiary structure for bovine CEL (PDB: 1aqlB) [1] served as the reference for the predicted human and zebrafish CEL tertiary structures, with a modeling range of residues 21 to 552. Theoretical isoelectric points and molecular weights for vertebrate CEL subunits were obtained using Expasy web tools (http://au.expasy.org/tools/pi_tool.html). SignalP 3.0 web tools were used to predict the presence and location of signal peptide cleavage sites (http://www.cbs.dtu.dk/services/SignalP/) for each of the predicted vertebrate CEL sequences [57]. The NetNGlyc 1.0 Server was used to predict potential N-glycosylation sites for vertebrate CEL subunits (http://www.cbs.dtu.dk/services/NetNGlyc/). 

### 2.3. Comparative Human (*CEL*) and Mouse (*Cel*) Tissue Expression

The UCSC Genome Browser (http://genome.ucsc.edu/) [4] was used to examine GNF1 Expression Atlas 2 data using various expression chips for human *CEL* and mouse *Cel *genes, respectively, (http://biogps.gnf.org/) [3]. Gene array expression “heat maps” were examined for comparative gene expression levels among human and mouse tissues showing high (red), intermediate (black), and low (green) expression levels. 

### 2.4. Phylogenetic Studies and Sequence Divergence

Alignments of vertebrate CEL and human, mouse, and nematode CES-like (carboxylesterase) protein sequences were assembled using BioEdit v.5.0.1 and the default settings [58]. Alignment ambiguous regions were excluded prior to phylogenetic analysis yielding alignments of 480 residues for comparisons of sequences (Table 1). Evolutionary distances were calculated using the Kimura option [59] in TREECON [60]. Phylogenetic trees were constructed from evolutionary distances using the neighbor-joining method [61] and rooted with the nematode CES sequence. Tree topology was reexamined by the boot-strap method (100 bootstraps were applied) of resampling, and only values that were highly significant (≥90) are shown [62].

## 3. Results and Discussion

### 3.1. Alignments of Human and Other Vertebrate CEL Subunits

**Figure 1:**
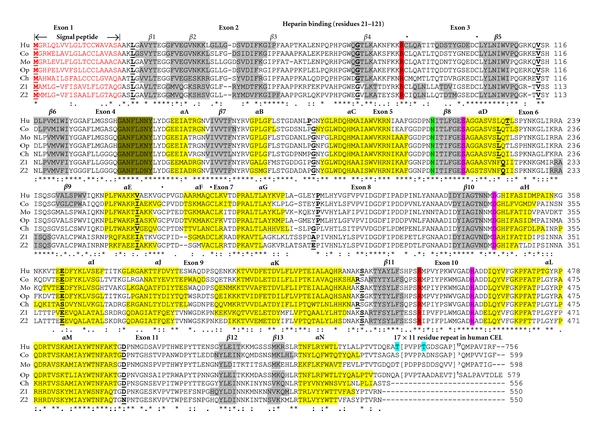
Amino acid sequence alignments for human and other vertebrate CEL subunits. See Table 1 for sources of CEL sequences; * shows identical residues for CEL subunits;: similar alternate residues;. dissimilar alternate residues; N-Signal peptide residues are in red; N-glycosylation residues at 207NIT (human CEL) are in green; active site (AS) triad residues Ser, Asp, and His are in pink; O-glycosylation sites are in blue; disulfide bond Cys residues for human CEL (•); essential arginines which contribute to bile-salt binding are in red; helix (human CEL or predicted helix); sheet (human CEL) or predicted sheet; bold font shows known or predicted exon junctions; exon numbers refer to human CEL gene; CEL “loop” covering the active site (human CEL residues 136–143) are in green; Hu-human CEL; Co-cow CEL; Mo-mouse CEL; Op-opossum CEL; Ch-chicken CEL; Z1-zebrafish CEL1; Z2-zebrafish CEL2.

**Figure 2:**
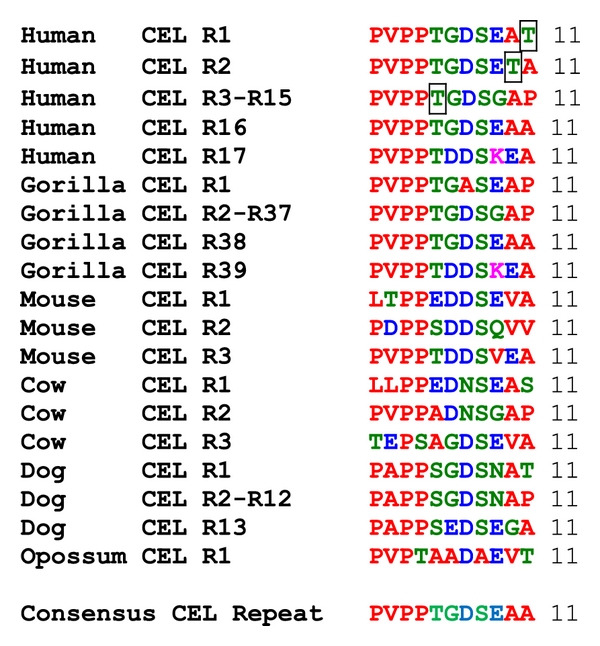
Amino acid alignments for C-terminal 11-residue repeat sequences for mammalian CEL subunits. Hydrophobic amino acid residues are shown in red; hydrophilic residues in green; acidic residues in blue; basic residues in pink; (squared T) refers to known O-glycosylation sites for human CEL; R refers to repeat number. P-proline; V-valine; T-threonine; G-glycine; D-aspartate; E-glutamate; S-serine; A-alanine; K-lysine; N-asparagine; note consistent PVPP start sequences.

The deduced amino acid sequences for opossum (*Monodelphis domestica*) and chicken (*Gallus gallus*) CEL subunits and for zebrafish (*Danio rerio*) CEL1 and CEL2 subunits are shown in Figure 1 together with the previously reported sequences for human (*Homo sapiens*) [7, 39], mouse (*Mus musculus*) [22], and bovine (*Bos taurus*) [27, 63] CEL subunits (Table 1). Alignments of the human and other vertebrate CEL subunits examined in this figure showed between 56–80% sequence identities, suggesting that these are products of the same family of genes and proteins (Table 2). The amino acid sequence for human CEL contained 756 residues whereas other vertebrate CEL subunits contained fewer amino acids: 599 residues (cow), 598 residues (mouse), 579 residues (opossum), 556 residues (chicken), and 550 residues for zebrafish CEL1 and CEL2 (Figure 1; Table 1). These differences are predominantly explained by changes in the number of VNTR 11 residue repeats at the respective CEL C-termini, with human CEL containing 17 repeats, whereas bovine, mouse, and opossum CEL C-termini contained only 3, 3, and 1 repeats, respectively, while chicken and zebrafish CEL subunits exhibited no VNTR-like C-terminus sequences. Table 1 summarizes this feature among all of the vertebrate CEL sequences examined and shows that substantial numbers of C-terminus VNTR repeats were predominantly restricted to higher primates, especially gorilla (*Gorilla gorilla*) (39 repeats) [20], human (17 repeats) [17], and rhesus (*Macaca mulatta*) (15 repeats) CEL, whereas other mammalian CEL subunits usually contained 3 VNTR repeats, with the exception of the predicted dog (*Canis familiaris*) CELC-terminus, which contained 13 VNTR-repeat sequences. A comparison of the 11-residue repeat sequences for the mammalian CEL subunits examined showed the following consensus sequence: Pro-Val-Pro-Pro-Thr-Gly-Asp-Ser-Glu-Ala-Ala (Figure 2), for which the first 4 residues have been proposed to play a role in facilitating O-glycosylation at the 5th residue (Thr) position [10]. 

Several other key amino acid residues for mammalian CEL have been recognized (sequence numbers refer to human CEL) (Figure 1). These include the catalytic triad for the active site (Ser194; Asp320; His435) forming a charge relay network for substrate hydrolysis [10, 64]; the hydrophobic N-terminus signal peptide (residues 1–20) [7, 65]; disulfide bond forming residues (Cys84/Cys100 and Cys266/Cys277) [7, 66]; arginine residues (Arg83/Arg446) which contribute to bile-salt binding and activation [1, 36]; a heparin binding site (residues 21–121); as well as the 11-residue VNTR repeat (×17) at the CEL C-terminus (residues 562–756). Identical residues were observed for each of the vertebrate CEL subunits for the active site triad, disulfide bond forming residues and key arginine residues contributing to bile salt activation, however, the N-terminus 20-residue signal peptide underwent changes in sequence but retained predicted signal peptide properties (Figure 1; Table 1). The N-glycosylation site reported for human CEL at Asn207-Ile208-Thr209 [10] was retained for each of the 22 vertebrate CEL sequences examined, with the exception of platypus (*Ornithorhynchus anatinus*) CEL which contained two predicted N-glycosylation sites at Asn381-Val382-Thr383 and Asn548-Leu549-Thr550 (Table 3). Predicted N-glycosylation sites were also observed at other positions, including Asn381-Ile382-Thr383 for opossum (*Monodelphis domestica*) CEL; Asn270-Thr271-Thr272 and Asn381-Leu382-Thr383 for chicken (*Gallus gallus*) CEL; Asn270-Thr271-Thr272 for lizard (*Anolis carolensis*); Asn550-Val551-Thr552 for fugu (*Takifugu rupides*) CEL (Table 3). Given the reported role of the N-glycosylated carbohydrate group in contributing to the stability and maintaining catalytic efficiency of a related enzyme (carboxylesterase or CES1) [67], this property may be shared by the vertebrate CEL subunits as well, especially for those containing multiple predicted sites for N-glycosylation, such as chicken CEL, which contains three such sites.

### 3.2. Predicted Secondary and Tertiary Structures of Vertebrate CEL Subunits

Analyses of predicted secondary structures for vertebrate CEL sequences were compared with the previously reported secondary structure for bovine and human CEL [1, 68] (Figure 1). Similar *α*-helix *β*-sheet structures were observed for all of the vertebrate CEL subunits examined. Consistent structures were particularly apparent near key residues or functional domains including the *β*-sheet and *α*-helix structures near the active site Ser194 (*β*8/*α*D) and Asp320 (*β*10/*α*8) residues, and the N-glycosylation site at Asn207-Ile208-Thr209 (near *β*8) [69]. The single helix at the C-termini (*α*N) for the vertebrate CEL subunits was readily apparent, as were the five *β*-sheet structures at the N-termini of the CEL subunits (*β*1–*β*5). It is apparent from these studies that all of these CEL subunits have highly similar secondary structures.

**Figure 3:**
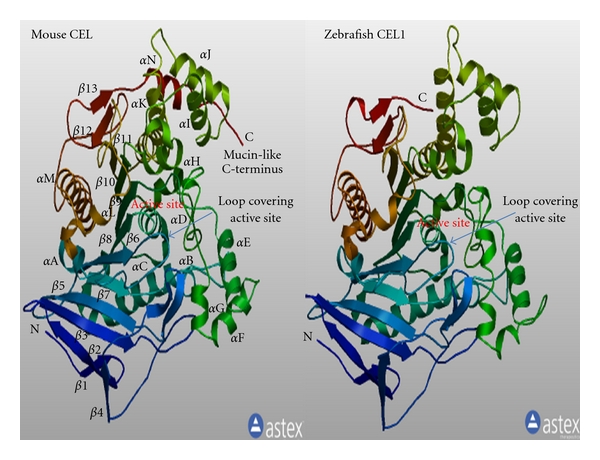
Predicted tertiary structures for mouse CEL and zebrafish CEL1 subunits. The predicted mouse CEL and zebrafish CEL1 3-D structures were obtained using the SWISS MODEL web site http://swissmodel.expasy.org/ and based on the reported structure for bovine CEL (PDB: 1aqlB) [1]; the rainbow color code describes the 3-D structures from the N- (blue) to C-termini (red color); N refers to amino terminus; C refers to carboxyl terminus; specific alpha helices (*α*A … *α*N) and beta sheets (*β*1 … *β*13) were identified, as well as the active site region and the “loop” covering the active site.

Figure 3 describes predicted tertiary structures for mouse CEL and zebrafish CEL1 protein sequences which showed significant similarities for these polypeptides with bovine [1, 36] and human CEL [68]. Identification of specific structures within the predicted mouse CEL and zebrafish CEL1 sequences was based on the reported structure for a truncated human CEL which identifies a sequence of twisted *β*-sheets interspersed with several *α*-helical structures [10, 68] which are typical of the alpha-beta hydrolase superfamily [40]. The active site CEL triad was centrally located which is similar to that observed in other lipases and esterases [40, 70, 71]. The major difference between CEL and other serine esterases is an apparent insertion at positions 139–146 (for human CEL) (Figure  1 of Supplementary Material available online at doi 10.1155/2011/781643) which appears to act as a surface loop that partially covers the opening to the catalytic triad and allows access to the active site by water soluble substrates by the truncated CEL [68]. This active site loop is also readily apparent in the predicted structures for mouse CEL and zebrafish CEL1. These comparative studies of vertebrate CEL proteins suggest that the properties, structures, and key sequences are substantially retained for all of the vertebrate sequences examined.

### 3.3. Predicted Gene Locations and Exonic Structures for Vertebrate *CEL* Genes

Table 1 summarizes the predicted locations for vertebrate *CEL* genes based upon BLAT interrogations of several vertebrate genomes using the reported sequences for human, gorilla, mouse, rat, and bovine CEL [6, 7, 20, 22–24, 27] and the predicted sequences for other vertebrate CEL proteins and the UCSC Genome Browser [4]. Human and mouse *CEL* genes were located on human chromosome 9 and mouse chromosome 2, which are distinct to the carboxylesterase (*CES* for human or *Ces* for mouse) gene family cluster locations in each case: on human chromosome 16 and mouse chromosome 8, respectively (Table 1; see [41]). The zebrafish (*Danio rerio*) genome showed evidence of tandem duplicated *CEL* genes, with predicted *CEL1* and *CEL2* genes being located about 7.3 kilobases apart on zebrafish chromosome 21 (Table 1). This is in contrast with many other gene duplication events during zebrafish evolution that have occurred predominantly by polyploidisation or duplication of large chromosomal segments rather than by tandem gene duplication [72]. 

Figure 1 summarizes the predicted exonic start sites for cow, opossum, chicken, and zebrafish *CEL* genes with each having 11 exons, in identical or similar positions to those reported for the human *CEL* and mouse *Cel* genes [17, 22, 23]. In contrast, human *CES1* [73, 74], *CES2*, *CES3* [75, 76], *CES4* [41], and CES5 [77, 78] genes contained 14, 12, 13, 14, and 13 exons, respectively, which are predominantly in distinct positions to those described for vertebrate *CEL* genes, with the exception of the last exon in each case (Figure  1 of Supplementary Material). Consequently, even though *CEL* and *CES* genes and proteins are members of the same serine hydrolase superfamily [10, 40], it is apparent that *CEL* is not a close relative of the *CES* gene family, for which at least five genes are clustered on a single chromosomes on human and mouse chromosomes and are more similar in gene structure to each other than they are to the *CEL* gene (Figure  1 of Supplementary Material; see [41]). 

**Figure 4:**
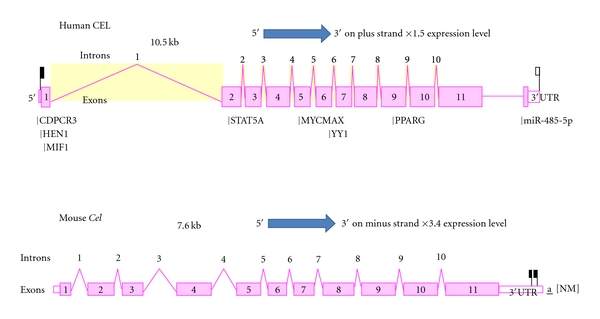
Gene structures for the human and mouse CEL genes. Derived from AceView website http://www.ncbi.nlm.nih.gov/IEB/Research/Acembly/ [2]; the major isoform variant is shown with capped 5′- and 3′- ends for the predicted mRNA sequences; introns and exons are numbered; the length of the mRNAs (as kilobases or kb) and comparative expression levels with the average gene are shown; a CpG island (CpG51); several predicted transcription factor binding sites; and a MiRNA485-5p binding site were identified for the human CEL gene; the direction for transcription is shown; 3′UTR refers to 3′-untranslated region.

Figure 4 illustrates the predicted structures of mRNAs for human and mouse *CEL *transcripts for the major transcript isoform in each case [2]. The transcripts were 10.5 and 7.6 kilobases in length, respectively, with 10 introns and 11 exons present for these *CEL *mRNA transcripts. The human *CEL* genome sequence contained a microRNA site (miR485-5p) located in the 3′-untranslated region and a CpG island (CpG51). The occurrence of the CpG island within the *CEL* gene may reflect a role in regulating gene expression [79] which may contribute to a higher than average gene expression level reported for human *CEL* (×1.5 times higher). Figure  2 of Supplementary Material shows a nucleotide sequence alignment diagram for the CpG51 region of the human *CEL* gene in comparison with several other mammalian and other vertebrate *CEL* genes. The Multiz alignment patterns observed demonstrated extensive sequence conservation for the CpG island which contains dinucleotide and trinucleotide repeat sequences in most genomes examined. 

The prediction of a microRNA (miRNA; miR485-5p) binding site in the 3′untranslated region of human *CEL* is also potentially of major significance for the regulation of this gene. MicroRNAs are small noncoding RNAs that regulate mRNA and protein expression and have been implicated in regulating gene expression during embryonic development [80]. Moreover, a recent study of a related miRNA gene (miR-375) has been recently shown to be selectively expressed in pancreatic islets and has been implicated both in the development of islets and the function of mature pancreatic beta cells [81]. A similar role may be played by miR485-5p with respect to the regulation of *CEL* expression during pancreatic beta cell development. Table  1 of Supplementary Material presents comparative nucleotide sequences for miR485-5p-like *CEL* gene regions for several vertebrate genomes which shows high levels of sequence identity, particularly among mammalian *CEL* miRNA target sites and suggests that this site has been predominantly conserved during vertebrate evolution, particularly by eutherian mammalian *CEL* genes. 

**Figure 5:**
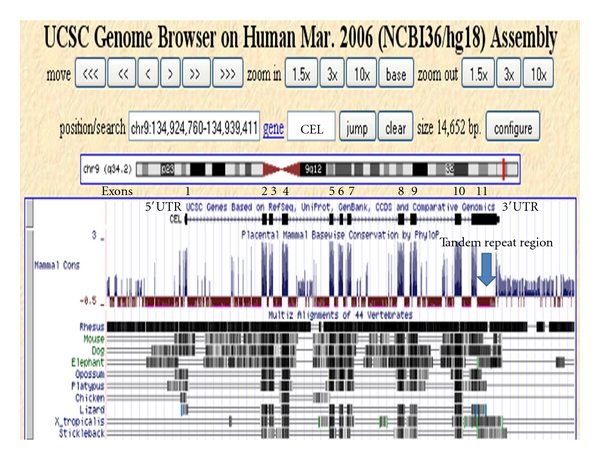
Comparative sequences for vertebrate 5′-flanking, 5′-untranslated, and coding regions for the *CEL* genes. Derived from the UCSC Genome Browser using the Comparative Genomics track to examine alignments and evolutionary conservation of *CEL* gene sequences; genomic sequences aligned for this study included primate (human and rhesus), nonprimate eutherian mammal (mouse, dog and elephant), a marsupial (opossum), a monotreme (platypus), bird (chicken), reptile (lizard), amphibian (frog), and fish (stickleback); conservation measures were based on conserved sequences across all of these species in the alignments which included the 5′flanking, 5′-untranslated (5′UTR), exons, introns, and 3′ untranslated (3′UTR) regions for the *CEL* gene; regions of sequence identity are shaded in different colors for different species; exons 1–11 are shown which are regions of conservation.

Figure 5 shows a UCSC Genome Browser Comparative Genomics track that shows evolutionary conservation and alignments of the nucleotide sequences for the human *CEL* gene, including the 5′-flanking, 5′-untranslated, intronic, exonic, and 3′untranslated regions of this gene, with the corresponding sequences for 10 vertebrate genomes, including 5 eutherian mammals (e.g., mouse, rat), a marsupial (opossum), a monotreme (platypus), and lower vertebrate genomes. Extensive conservation was observed among these genomic sequences, particularly for the rhesus *CEL* gene and for other eutherian mammalian genomes. In contrast with the eutherian mammalian genomes examined, other vertebrate genomes retained conserved sequences only within the 11 exonic *CEL* regions. It would appear that exonic *CEL *nucleotide sequences have been conserved throughout vertebrate evolution whereas only in eutherian mammalian genomes have other regions of the *CEL* gene been predominantly conserved. 

### 3.4. Comparative Human and Mouse *CEL* Tissue Expression

**Figure 6:**
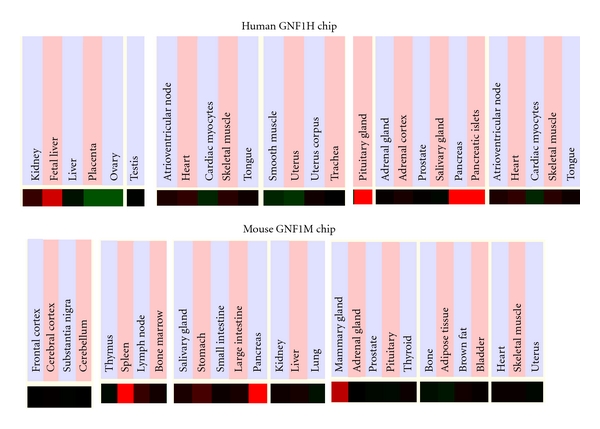
Comparative tissue expression for human and mouse *CEL *genes. Expression “heat maps” (GNF Expression Atlas 2 data) (http://biogps.gnf.org/) [3] were examined for comparative gene expression levels among selected human (GNF1H) and mouse (GNF1M) tissues for *CEL* showing high (red), intermediate (black), and low (green) expression levels. The results were derived from the human and mouse genome browsers (http://genome.ucsc.edu/) [4].

Figure 6 presents “heat maps” showing comparative gene expression for various human and mouse tissues obtained from GNF Expression Atlas Data using the GNF1H (human) and GNF1M (mouse) chips (http://genome.ucsc.edu/; http://biogps.gnf.org/) [3]. These data supported a high level of tissue expression for human *CEL*, particularly for the pancreatic islets, the pituitary gland, and fetal liver, which is consistent with previous reports for these genes (see [10]). High levels of *CEL* gene expression have also been reported for the human mammary gland where CEL plays a major role in lipid digestion in breast milk by neonates [6]. The localization of CEL within the human pituitary gland is of major interest as this enzyme also hydrolyzes ceramides [8], which suggests a possible role in the regulation of hormone secretion in both normal and adenomatous pituitary cells [16]. A high level of expression of CEL in mouse tissues was also observed (×3.4 times average expression) (Figure 4), particularly for the pancreas, mammary gland, and spleen (Figure 5), where similar metabolic roles for this enzyme in cholesterol ester, retinyl ester, and triglyceride hydrolysis and metabolism have been described [10]. Recent metabolic studies using *Cel^−^*/*Cel^−^* (knock-out) mice (or CELKO mice) have demonstrated that CEL is not an essential enzyme for these metabolic functions [82, 83], although CELKO neonatal mice exhibit an incomplete digestion of milk fat [9, 84], and in adult CELKO mice causes an elevation in reverse cholesterol transport (RCT) in adult animals [14]. The latter finding is potentially of major clinical significance for this enzyme, given that any increase in RCT and the associated increased biliary disposal of cholesterol may contribute to preventing atherosclerosis [85, 86]. 

### 3.5. Phylogeny and Divergence of Vertebrate *CEL* and Mammalian/Nematode *CES* Sequences

**Figure 7:**
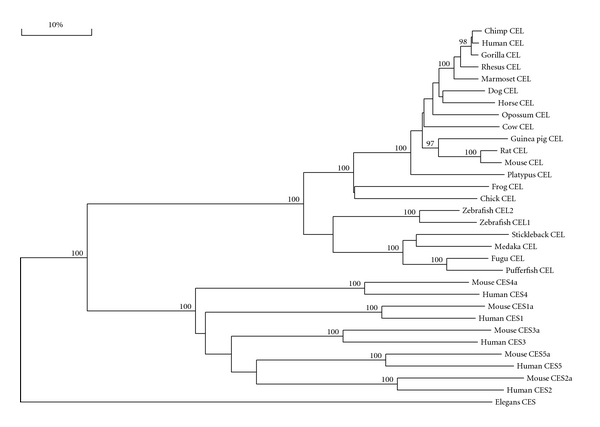
Phylogenetic tree of vertebrate CEL with human and mouse CES1, CES2, CES3, CES4, and CES5 amino acid sequences. The tree is labeled with the gene name and the name of the vertebrate. Note the major cluster for the vertebrate CEL sequences and the separation of these sequences from human and mouse CES1, CES2, CES3, CES4, and CES5 sequences. The tree is “rooted” with the CES sequence (T27C12) from a nematode (*Caenorhabditis elegans*). See Table 1 for details of sequences and gene locations. A genetic distance scale is shown (% amino acid substitutions). The number of times a clade (sequences common to a node or branch) occurred in the bootstrap replicates are shown. Only replicate values of 90 or more which are highly significant are shown with 100 bootstrap replicates performed in each case.

A phylogenetic tree (Figure 7) was calculated by the progressive alignment of human and other vertebrate CEL amino acid sequences with human and mouse CES1, CES2, CES3, CES4, and CES5 sequences. The phylogram was “rooted” with a nematode CES sequence and showed clustering of the CEL sequences which were distinct from the human and mouse CES families. In addition, the zebrafish CEL1 and CEL2 sequences showed clustering within the fish CEL sequences examined, which is consistent with these genes being products of a recent duplication event during teleost fish evolution. Overall, these data suggest that the vertebrate *CEL* gene arose from a gene duplication event of an ancestral *CES*-like gene, resulting in at least two separate lines of gene evolution for *CES*-like and *CEL*-like genes. This is supported by the comparative biochemical and genomic evidence for vertebrate *CEL* and *CES*-like genes and encoded proteins, which share several key features of protein and gene structure, including having similar alpha-beta hydrolase secondary and tertiary structures [10, 40, 41, 71, 78] (Figure  1 of Supplementary Material). 

In conclusion, the results of the present study indicate that vertebrate *CEL* genes and encoded CEL enzymes represent a distinct alpha-beta hydrolase gene and enzyme family which share key conserved sequences and structures that have been reported for the human *CES* gene families. CEL is a major triglyceride, cholesterol ester and vitamin ester hydrolytic enzyme contained within exocrine pancreatic and lactating mammary gland secretions and is also localized in plasma where it contributes to chylomicron assembly and secretion, in the selective uptake of cholesteryl esters in HDL in the liver and in reverse cholesterol transport, including biliary disposal of cholesterol. Bioinformatic methods were used to predict the amino acid sequences, secondary and tertiary structures and gene locations for *CEL* genes, and encoded proteins using data from several vertebrate genome projects. A proline-rich and O-glycosylated 11-amino acid C-terminal repeat sequence (VNTR) previously reported for human and other higher primate CEL proteins was also observed for other eutherian mammalian CEL sequences examined. Opossum CEL, however, contained a single C-terminal copy of this sequence while CEL proteins from lower vertebrates lacked the VNTR sequence. Evidence is presented for tandem duplicated *CEL* genes for the zebrafish genome. Vertebrate CEL protein subunits shared 53–97% sequence identities and exhibited sequence alignments and identities for key CEL amino acid residues as well as extensive conservation of predicted secondary and tertiary structures with those previously reported for human CEL. Phylogenetic analyses demonstrated the relationships and potential evolutionary origins of the vertebrate *CEL* family of genes which were related to a nematode carboxylesterase (*CES*) gene and five mammalian *CES* gene families. These studies indicated that *CEL* genes have apparently appeared early in vertebrate evolution prior to the teleost fish common ancestor more than 500 million years ago [87].

## Supplementary Material

Supplementary Figure 1 shows that one of the major differences between vertebrate CEL sequences and those of other serine esterases (such as the carboxylesterases CES1-CES6) is an apparent insertion at positions 139–146 which appears to act as a surface loop that partially covers the opening to the catalytic triad and allows access to the active site by water soluble substrates by the truncated CEL. This active site loop is also readily apparent in the predicted structures for mouse CEL and zebrafish CEL1.
Supplementary Figure 2 shows a comparative nucleotide sequence alignment diagram for the CpG51 region of the human *CEL* gene in comparison with several other mammalian and other vertebrate *CEL* genes; derived from the UCSC Genome Browser using the Comparative Genomics track to examine alignments and evolutionary conservation of *CEL* gene sequences for the CpG51 region containing dinucleotide and trinucleotide repeats; regions of sequence identity are shaded from black to a lighter color according to the degree of identity. The Multiz alignment patterns observed demonstrated extensive sequence conservation for the CpG island which contains dinucleotide and trinucleotide repeat sequences in most genomes examined.
Supplementary Table presents comparative nucleotide sequences for miR485-5p like *CEL* gene regions for several vertebrate genomes which shows high levels of sequence identity, particularly among mammalian *CEL* miRNA target sites and suggests that this site has been predominantly conserved during vertebrate evolution, particularly by eutherian mammalian *CEL* genes.

## Figures and Tables

**Table 1:** Vertebrate *CEL*, human and mouse carboxylesterase (*CES1–5*) and nematode *CES* genes and subunits.

Vertebrate	Species	Gene *CEL *	RefSeq ID Prediction^1,2^	GenBank ID	Chromosome location	Exons (strand)	Gene size (bps)	UNIPROT ID	Amino acids	Subunit MW	pI	No. of repeats
Human	*Homo Sapiens *	*CEL*	NM_001807	BC042510	9:134, 927, 202-134, 936, 969	11 (+ve)	9,768	P19835	756	79,667	5.1	17
Gorilla	*Gorilla gorilla*	*CEL*	sc^4^6591^2^	³	sc6591^4^:90, 194-97, 985	³	7,791	Q9N1D1	998	101,026	4.5	39
Rhesus	*Macaca mulatta*	*CEL*	chr15.6.020²	³	15:5, 562, 165-5, 571, 662	11 (−ve)	9,498	³	731	78,302	4.8	15
Mouse	*Mus musculus*	*CEL*	NM_009885	BC006872	2:28, 411, 584-28, 418, 882	11 (−ve)	7,543	Q64285	598	65,666	5.9	3
Rat	*Rattus norvegicus*	*CEL*	NM_016997	M15893	3:7, 541, 329-7, 549, 228	11 (−ve)	7,900	P07882	612	67,052	5.3	4
Guinea pig	*Cavia porcellus*	*CEL*	sc^4^27.013.1^2^	³	sc27^4^:846, 072-851, 868	11 (−ve)	5,797	³	598	65,174	5.4	3
Cow	*Bos taurus*	*CEL*	NP_001013601	BC149530	11:106, 810, 242-106, 819, 101	11 (+ve)	8,762	P30122	599	65,598	5.2	3
Horse	*Equus caballus*	*CEL*	chr25.415.1²	³	25:35, 342, 417-35, 350, 330	11 (+ve)	7,914	³	599	65,310	5.3	3
Dog	*Canis familiaris*	*CEL*	chr9.55.011²	³	9:54, 678, 403-54, 686, 294	11 (−ve)	7,892	³	709	75,920	5.4	13
Opossum	*Monodelphis domestica*	*CEL*	chr1.10.178²	³	1:460, 155, 249-460, 171, 513	11 (+ve)	16,265	³	579	63,553	6.4	1
Platypus	*Ornithorhynchus anatinus*	*CEL*	ENSOANT23206²	³	Ultra81^4^:471, 532-485, 254	11 (+ve)	13,723	³	557	61,672	7.1	0
Chicken	*Gallus gallus*	*CEL*	NP_001013015*¹*	BX950478	17:7, 428, 231-7, 433, 490	11 (+ve)	5,260	³	556	61,191	6.6	0
Lizard	*Anolis carolinensis*	*CEL*	ENSACAT4924²	³	353^4^:801, 542-811, 604	11 (+ve)	10,063	³	553	61,438	6.1	0
Frog	*Xenopus tropicalis*	*CEL*	NP_001120027*¹*	³	191^4^:155, 996-171, 454	11 (−ve)	15,459	³	552	60,653	6.2	0
Zebrafish	*Danio rerio*	*CEL1*	NM_199607	BC079529	21:4, 281, 250-4, 291, 233	11 (−ve)	9,984	³	550	60,521	6.0	0
Zebrafish	*Danio rerio*	*CEL2*	AAH65887*⁵*	BC096893	21:4, 298, 674-4, 307, 810	11 (−ve)	9,137	³	550	60,425	6.1	0
Stickleback	*Gasterosteus aculeatus*	*CEL*	ENSGACT24004²	³	XIV:11, 357, 393-11, 361, 602	11 (−ve)	4,210	³	555	60,963	5.7	0
Medaka	*Oryzias latipes*	*CEL*	ENSORLT18090²	³	12:26, 977, 209-26, 983, 642	11 (+ve)	6,434	³	552	60,329	5.8	0
Fugu	*Takifugu rubripes*	*CEL*	ENSTRUG2423²	³	sc370^4^:307, 466, 106-307, 469, 087	11 (−ve)	2,982	³	554	61,498	6.7	0
Pufferfish	*Tetraodon nigroviridis*	*CEL*	CAF94246	CR654166	Un^5^:12, 727, 719-12, 730, 519	11 (−ve)	4,210	³	555	60,859	5.7	0
		*CES*										
Human	*Homo Sapiens *	*CES1*	NM_001025195	L07765	16:54, 394, 465-54, 424, 468	14 (−ve)	30,004	P23141	567	62,521	6.2	³
Mouse	*Mus musculus*	*CES1a*	NM_001013764	BC089371	8:95, 544, 116-95, 572, 091	14 (−ve)	27,979	Q5FWH4	563	61,744	5.1	³
Human	*Homo Sapiens *	*CES2*	NM_003869	BC032095	16:65, 527, 040-65, 535, 426	12 (+ve)	8,387	O00748	559	61,807	5.7	³
Mouse	*Mus musculus*	*Ces2a*	NM_133960	BC024491	8:107, 257, 972-107, 265, 313	12 (+ve)	7,342	Q8QZR3	558	61,940	5.7	³
Human	*Homo Sapiens *	*CES3*	NM_024922	BC053670	16:65, 552, 712-65, 564, 450	13 (+ve)	11,739	Q9H6X7	571	62,282	5.4	³
Mouse	*Mus musculus*	*Ces3a*	NM_198672	AK138932	8:107, 572, 572-107, 582, 000	13 (+ve)	21,512	Q63880	554	61,510	5.8	³
Human	*Homo Sapiens *	*CES4A*	NM_173815	BC166638	16:65, 580, 177-65, 600, 543	14 (+ve)	20,367	Q5XG92	561	60,366	9.4	³
Mouse	*Mus musculus*	*Ces4a*	NM_146213	BC026374	8:107, 655, 852-107, 673, 417	14 (+ve)	17,566	³	563	62,123	8.8	³
Human	*Homo Sapiens *	*CES5A*	NM_001143685	BC039073	16:54, 437, 867-54, 466, 634	13 (−ve)	27,768	Q6NT32	575	63,936	6.0	³
Mouse	*Mus musculus*	*Ces5a*	NM_001003951	AB186393	8:96, 038, 095-96, 059, 607	13 (+ve)	21,512	Q8R0W5	575	64,167	5.5	³
Nematode	*Caenorhabditis elegans*	*T28C12*	NM_072212	AAB66159	V:6, 323, 188-6, 326, 373	9 (+ve)	3,186	Q4LDP0	658	74,736	9.2	³

RefSeq refers to the NCBI reference sequence; *¹*predicted NCBI sequence; ²predicted UCSC Genome Browser sequence; ³not available; ^4^gene scaffold ID; ^5^unidentified chromosome; pI refers to isoelectric point; bps refers to base pairs of nucleotide sequence; repeats number refers to the number of VNTR sequences at the CEL C-terminus.

**Table 2:** Percentage identities for vertebrate CEL subunit amino acid sequences.

Vertebrate	Human	Chimp	Gorilla	Rhesus	Marmoset	Mouse	Rat	G pig	Cow	Horse	Dog	Opossum	Platypus	Chick	Frog	Zfish 1	Zfish 2	Pufferfish	Medaka	Fugu
Human	100	97	97	93	92	80	80	78	78	84	86	84	75	65	65	56	58	53	54	54
Chimp	97	100	97	93	91	80	80	78	79	84	86	83	75	65	65	56	58	53	54	54
Gorilla	97	97	100	93	93	80	80	78	79	84	86	83	75	65	65	56	58	54	55	54
Rhesus	93	93	93	100	92	80	80	79	80	85	87	84	76	65	66	57	58	55	55	55
Marmoset	92	91	93	92	100	81	82	79	80	85	87	85	76	66	67	57	59	53	56	54
Mouse	80	80	80	80	81	100	93	80	78	77	80	77	72	63	63	57	59	53	55	53
Rat	80	80	80	80	82	93	100	82	79	78	79	78	73	64	63	58	60	55	56	54
Guinea pig	78	78	78	79	79	80	82	100	78	79	78	77	73	64	65	57	59	54	56	55
Cow	78	79	79	80	80	78	79	78	100	81	81	78	73	66	65	57	58	56	57	56
Horse	84	84	84	85	85	77	78	79	81	100	85	81	75	67	65	58	59	55	56	55
Dog	86	86	86	87	87	80	79	78	81	85	100	83	77	66	64	58	59	54	55	55
Opossum	84	83	83	84	85	77	78	77	78	81	83	100	77	65	65	57	58	55	55	54
Platypus	75	75	75	76	76	72	73	73	73	75	77	77	100	66	62	55	57	54	54	55
Chick	65	65	65	65	66	63	64	64	66	67	66	65	66	100	67	60	62	56	58	56
Frog	65	65	65	66	67	63	63	65	65	65	64	65	62	67	100	58	57	57	58	56
Zfish 1	56	56	56	57	57	57	58	57	57	58	58	57	55	60	58	100	86	64	63	64
Zfish 2	58	58	58	58	59	59	60	59	58	59	59	58	57	62	57	86	100	62	66	63
Pufferfish	53	53	54	55	53	53	55	54	56	55	54	55	54	56	57	64	62	100	71	83
Medaka	54	54	55	55	56	55	56	56	57	56	55	55	54	58	58	63	66	71	100	72
Fugu	54	54	54	55	54	53	54	55	56	55	55	54	55	56	56	64	63	83	72	100

Zfish refers to zebrafish (*Danio rerio*) CEL; Gpig refers to guinea pig CEL (*Cavia porcellus*).

**Table 3:** Known or predicted N-glycosylation sites for vertebrate CEL subunits.

Vertebrate	Site 1	Site 2	Site 3	Site 4	Site 5	Site 6	No of sites
CEL							
Human	**210NIT**						1
Chimp	**207NIT**						1
Gorilla	**210NIT**				518NGS		1
Rhesus	**207NLT**				515NGS		1
Marmoset	**207NIT**				515NGS		1
Mouse	**207NIT**		325NNT				1
Rat	**207NIT**						1
Guinea pig	**207NIT**						1
Cow	**207NIT**			381NAT			1
Horse	**207NIT**			381NAT	515NSS		1
Dog	**207NIT**			381NST		567NAT	1
Opossum	**207NIT**			**381NIT**			2
Platypus				**381NVT**		**548NLT**	2
Chicken	**207NIT**	**270NTT**		**381NLT**			3
Lizard	**207NIT**	**270NTT**					2
Frog	**207NIT**						1
Zebrafish 1	**204NIT**						1
Zebrafish 2	**204NIT**						1
Pufferfish	**204NIT**						1
Fugu	**204NIT**					**550NVT**	2
Stickleback	**205NIT**						1
Medaka	**204NIT**						1

The identified N-glycosylation site is for human CEL (see [10]). Amino acid residues are shown for known or predicted N-glycosylation sites: N-Asn; A-Ala; T-Thr; S-Ser; M-Met; L-Leu; D-Asp; G-Gly; F-Phe; I-Ile; V-Val; sites with high probabilities for N-glycosylation are written in **bold face**.
