# Supplementary material for: Comparative Structures and Evolution of Vertebrate Carboxyl Ester Lipase (CEL) Genes and Proteins with a Major Role in Reverse Cholesterol Transport
Source: Cholesterol. 2011 Nov 21;2011:781643. doi: 10.1155/2011/781643 (PMC3227413; doi:10.1155/2011/781643)
Supplement: Supplementary file 1 — Supplementary Figure 1 shows that one of the major differences between vertebrate CEL sequences and those of other serine esterases (such as the carboxylesterases CES1-CES6) is an apparent insertion at positions 139–146 which appears to act as a surface loop that partially covers the opening to the catalytic triad and allows access to the active site by water soluble substrates by the truncated CEL. This active site loop is also readily apparent in the predicted structures for mouse CEL and zebrafish CEL1. Supplementary Figure 2 shows a comparative nucleotide sequence alignment diagram for the CpG51 region of the human CEL gene in comparison with several other mammalian and other vertebrate CEL genes; derived from the UCSC Genome Browser using the Comparative Genomics track to examine alignments and evolutionary conservation of CEL gene sequences for the CpG51 region containing dinucleotide and trinucleotide repeats; regions of sequence identity are shaded from black to a lighter color according to the degree of identity. The Multiz alignment patterns observed demonstrated extensive sequence conservation for the CpG island which contains dinucleotide and trinucleotide repeat sequences in most genomes examined. Supplementary Table presents comparative nucleotide sequences for miR485-5p like CEL gene regions for several vertebrate genomes which shows high levels of sequence identity, particularly among mammalian CEL miRNA target sites and suggests that this site has been predominantly conserved during vertebrate evolution, particularly by eutherian mammalian CEL genes. [file 781643.f1.docx]

**Exon 1** **Signal peptide Exon 2 Exon 3 • |🡨CES insert🡪|**

HuCEL **M**-------**LTMGRLQLVVLGLTCCWAVASA**AK**L**GAVYTEGGFVEGVNKKLGLLG-DSVDIFKGIPFAAP---TKALENPQPHPGWQ**G**TLKAKNFKKRCLQATIT--------------QD 95

RaCEL **M**----------**GRLEVLFLGLTCCLAAACA**AK**L**GAVYTEGGFVEGVNKKLSLLGGDSVDIFKGIPFAT----AKTLENPQRHPGWQ**G**TLKATDFKKRCLQATIT--------------QD 92

HuCES1 **M**----------**WLRAFILATLSASAAWG**HPSSPPVVDTVHGKVLGK-FVSLEGFAQPVAIFLGIPFAKPPLGPLRFTPPQPAEPWSFVKNATSYPPM**C**TQDPKAGQLLSELFTNRKENIP 109

HuCES2 **M**-**RLHRLR**-**ARLSAVACGLLLLLVRGQG**QDSASPIRTTHTGQVLGS-LVHVKGANAGVQTFLGIPFAKPPLGPLRFAPPEPPESWSGVRDGTTHPAM**C**LQDLTA--VESEFLSQFNMTFP 115

HuCES3 MERAVRVESGVLVGVVCLLLACPATAT**G**PEVAQPEVDTTLGRVRGR-QVGVKGTDRLVNVFLGIPFAQPPLGPDRFSAPHPAQPWEGVRDASTAPPM**C**LQDVES--MNSSRF-VLNGKQQ 116

HuCES4 **M**-------**RWILCWSLTLCLMAQTALG**ALHTKRPQVVTKYGTLQGK-QMHVG--KTPIQVFLGVPFSRPPLGILRFAPPEPPEPWKGIRDATTYPPG**C**LQESWG--QLASMYVSTRERYK 108

HuCES5 **M**-**SGNWVHPGQILIWAIWVLAAPTK**--**G**PSAEGPQRNTRLGWIQGK-QVTVLGSPVPVNVFLGVPFAAPPLGSLRFTNPQPASPWDNLREATSYPNL**C**LQNSEWLLLDQHML---KVHYP 113

. * * : * : * *:**: : *. *. ... * *

• **Exon 4 Exon 5**

HuCEL STYGDEDCLYLNIWVPQGRKQ**V**SRDLPVMIWIYGGAFLMGSGHGANFLNNYLYDGEEIATRGNVIVVTFNYRVGPLGFLSTGDANL**P**GNYGLRDQHMAIAWVKRNIAAFGGDPNNITLFG 215

RaCEL DTYGQEDCLYLNIWVPQGRKQ**V**SHDLPVMVWIYGGAFLMGSGQGANFLKNYLYDGEEIATRGNVIVVTFNYRVGPLGFLSTGDANLP**G**NFGLRDQHMAIAWVKRNIAAFGGDPDNITIFG 212

HuCES1 -LKLSEDCLYLNIYTP-ADLTKKNRLP**V**MVWIHGGGLMVGAAS--------TYDGLALAAHENVVVVTIQYRLGIWGFF**S**TGDEHSRGNWGHLDQVAALRWVQDNIASFGGNPGSVTIFG 219

HuCES2 SDSMSEDCLYLSIYTP-AHSHEGSNLP**V**MVWIHGGALVFGMAS--------LYDGSMLAALENVVVVIIQYRLGVLGFF**S**TGDKHATGNWGYLDQVAALRWVQQNIAHFGGNPDRVTIFG 226

HuCES3 IFSVSEDCLVLNVYSP-AEVPAGSGRP**V**MVWVHGGALITGAAT--------SYDGSALAAYGDVVVVTVQYRLGVLGFF**S**TGDEHAPGNQGFLDVVAALRWVQENIAPFGGDLNCVTVFG 227

HuCES4 WLRFSEDCLYLNVYAP-ARAPGDPQLP**V**MVWFPGGAFIVGAAS--------SYEGSDLAAREKVVLVFLQHRLGIFGFL**S**TDDSHARGNWGLLDQMAALRWVQENIAAFGGDPGNVTLFG 219

HuCES5 KFGVSEDCLYLNIYAP-AHADTGSKLP**V**LVWFPGGAFKTGSAS--------IFDGSALAAYEDVLVVVVQYRLGIFGFF**T**TWDQHAPGNWAFKDQVAALSWVQKNIEFFGGDPSSVTIFG 224

.**** *.:: * **::*. **.: * . ::* :*: .*::* .::*:* **::* * : ** . * *: **: ** ***: . :*:**

**Exon 6** **• • Exon 7 Exon 8**

HuCEL ESAGGASVSL**Q**TLSPYNKGLIRRAISQSGVALSPWVIQK--NPLFWAKK**V**AEKVGCPVGDAARMAQCLKVTDPRALTLAYKVP----LAGLEY**P**MLHYVGFVPVIDGDFIPADPINLYAN 329

RaCEL ESAGAASVS**L**QTLSPYNKGLIRRAISQSGVALSPWAIQE--NPLFWAKT**I**AKKVGCPTEDTAKMAGCLKITDPRALTLAYRLP----LKSQEY**P**IVHYLAFIPVVDGDFIPDDPINLYDN 326

HuCES1 ESAGGESVSVL**V**LSPLAKNLFHRAISESGVALTSVLVKKG-DVKPLAE**Q**IAITAGCKTTTSAVMVHCLRQKTEEELLETTLKM**K**FLSLDLQGDPRE**S**QPLLGTVIDGMLLLKTPEELQAE 338

HuCES2 ESAGGTSVSSLVVSPISQGLFHGAIMESGVALLPGLIAS--SADVIST**V**VANLSACDQVDSEALVGCLRGKSKEEILAINK**P**---------------FKMIPGVVDGVFLPRHPQELLAS 329

HuCES3 GSAGGSIISGL**V**LSPVAAGLFHRAITQSGVITTPGIIDS--HPWPLA**Q**KIANTLACSSSSPAEMVQCLQQKEGEELVLSKKL**K**--------------NTIYPLTVDGTVFPKSPKELLKE 331

HuCES4 QSAGAMSISGL**M**MSPLASGLFHRAISQSGTALFRLFITS--NPLKVAK**K**VAHLAGCNHNSTQILVNCLRALSGTKVMRVSNKM**R**FLQLNFQRDPEE**I**IWSMSPVVDGVVIPDDPLVLLTQ 337

HuCES5 ESAGAISVSSL**I**LSPMAKGLFHKAIMESGVAIIPYLEAHDYEKSED**L**QVVAHFCGNNASDSEALLRCLRTKPSKELLTLS**Q**KT---------------KSFTRVVDGAFFPNEPLDLLSQ 329

***. :* :** .*:: ** :**. :* . . : *:: : .:** .: * * .

**Exon 9**

HuCEL --AADIDYIAGTNNMDGHIFASIDMPAINKGNKKVTE**E**DFYKLVSEFTITKGLRGAKTTFDVYTESWAQ---DPSQENKKKTVVDFETDVLFLVPTEIALAQHRANAK**S**AKTYAYLFSHP 444

RaCEL --AADIDYLAGINDMDGHLFATVDVPAIDKAKQDVTE**E**DFYRLVSGHTVAKGLKGTQATFDIYTESWAQ---DPSQENMKKTVVAFETDILFLIPTEMALAQHRAHAK**S**AKTYSYLFSHP 441

HuCES1 RNFHTVPYMVGINKQEFGWLIPM**Q**LMSYPLSEGQLDQKTAMSLLWKSYPLV**C**IA--KELIPEATEKYLG--GTDDTVKKKDLFLDLIADVMFGVPSVIVARNHR**D**AGAPTYMYEFQYRPS 454

HuCES2 ADFQPVPSIVGVNNNEFGWLIPKVMRIYDT-QKEMDREASQAALQKMLTLL**M**LP--PTFGDLLREEYIG--DNGDPQTLQAQFQEMMADSMFVIPALQVA-HFQC**S**RAPVYFYEFQHQPS 443

HuCES3 KPFHSVPFLMGVNNHEFSWLIPR**G**WGLLDTMEQ-MSREDMLAISTPVLTSL**D**VP--PEMMPTVIDEYLG--SNSDAQAKCQAFQEFMGDVFINVPTVSFSRYLR**D**SGSPVFFYEFQHRPS 446

HuCES4 GKVSSVPYLLGVNNLEFNWLLPY**I-**MKFPLNRQAMRKETITKMLWSTRTLL**N**IT--KEQVPLVVEEYLDNVNEHDWKMLRNRMMDIVQDATFVYATLQTAHYHR**D**AGLPVYLYEFEHHAR 454

HuCES5 KAFKAIPSIIGVNNHECGFLLPM**K**-----EAPEILSGSNKSLALHLIQNIL**H**IP--PQYLHLVANEYFH--DKHSLTEIRDSLLDLLGDVFFVVPALITARYHR**D**AGAPVYFYEFRHRPQ 440

: : * *. : : . : . : : : . . : * : .: . * : .

**Exon 10 Exon 11**

HuCEL SRMPVYPKWVGADHADDIQYVFGKPFATPTG-------YRPQDRTVSKAMIAYWTNFAKTG**D**PNMGDSAVPTHWEPYTTENSGYLEITKKMGSSSMKRSLRTNFLRYWTLTYLALPTVTD 557

RaCEL SRMPIYPKWMGADHADDLQYVFGKPFATPLG-------YRAQDRTVSKAMIAYWTNFAKSG**D**PNMGNSPVPTHWYPYTTENGNYLDINKKITSTSMKEHLREKFLKFWAVTFEMLPTVVG 554

HuCES1 FSSDMKPKTVIGDHGDELFSVFGAPFLK**E**G--------ASEEEIRLSKMVMKFWANFARNG**N**PNGEG---LPHWPEYNQKE-GYLQIGANTQAA---QKLKDKEVAFWTNLFAKKAVEK- 558

HuCES2 WLKNIRPPHMKADHGDELPFVFRSFFGGN------YIKFTEEEEQLSRKMMKYWANFARNG**N**PNGEG---LPHWPLFDQEE-QYLQLNLQPAVG---RALKAHRLQFWKKALPQKIQELE 550

HuCES3 SFAKIKPAWVKADHGAEGAFVFGGPFLMDESSRLAFPEATEEEKQLSLTMMAQWTHFARTG**D**PNSKA---LPPWPQFNQAE-QYLEINPVPRAG---QKFREAWMQFWSETLPSKIQQWH 559

HuCES4 -GIIVKPRTDGADHGDEMYFLFGGPFATG--------LSMGKEKALSLQMMKYWANFARTG**N**PNDGN---LPCWPRYNKDE-KYLQLDFTTRVG---MKLKEKKMAFWMSLYQSQRPEKQ 558

HuCES5 CFEDTKPAFVKADHADEVRFVFGGAFLKGD--IVMFEGATEEEKLLSRKMMKYWATFARTG**N**PNGND---LSLWPAYNLTE-QYLQLDLNMSLG---QRLKEPRVDFWTSTIPLILSASD 551

* .**. : :* * :: :* :: *: **:.*:** . * : : **:: :: : :*

HuCEL QEAT-[VNTN] repeats x17 756 extended (x17) O-glycosylated mucin sequence at C-terminus

RaCEL DHT- [VNTN] repeats x 4 612 extended (x4) O-glycosylated mucin sequence at C-terminus

HuCES1 PPQTE---**HIEL**------------ 567 **microsomal targeting sequence**

HuCES2 EPEER---**HTEL**------------ 559 **microsomal targeting sequence**

HuCES3 QKQKNRKA**QEDL**------------ 571 **microsomal targeting sequence**

HuCES4 RQF--------------------- 561

HuCES5 MLHSPLSSLTFLSLLQPFFFFCAP 575
