# Supplementary figures and images for: Comparative Structures and Evolution of Vertebrate Carboxyl Ester Lipase (CEL) Genes and Proteins with a Major Role in Reverse Cholesterol Transport
Source: Cholesterol. 2011 Nov 21;2011:781643. doi: 10.1155/2011/781643 (PMC3227413; doi:10.1155/2011/781643)

## Slide 1
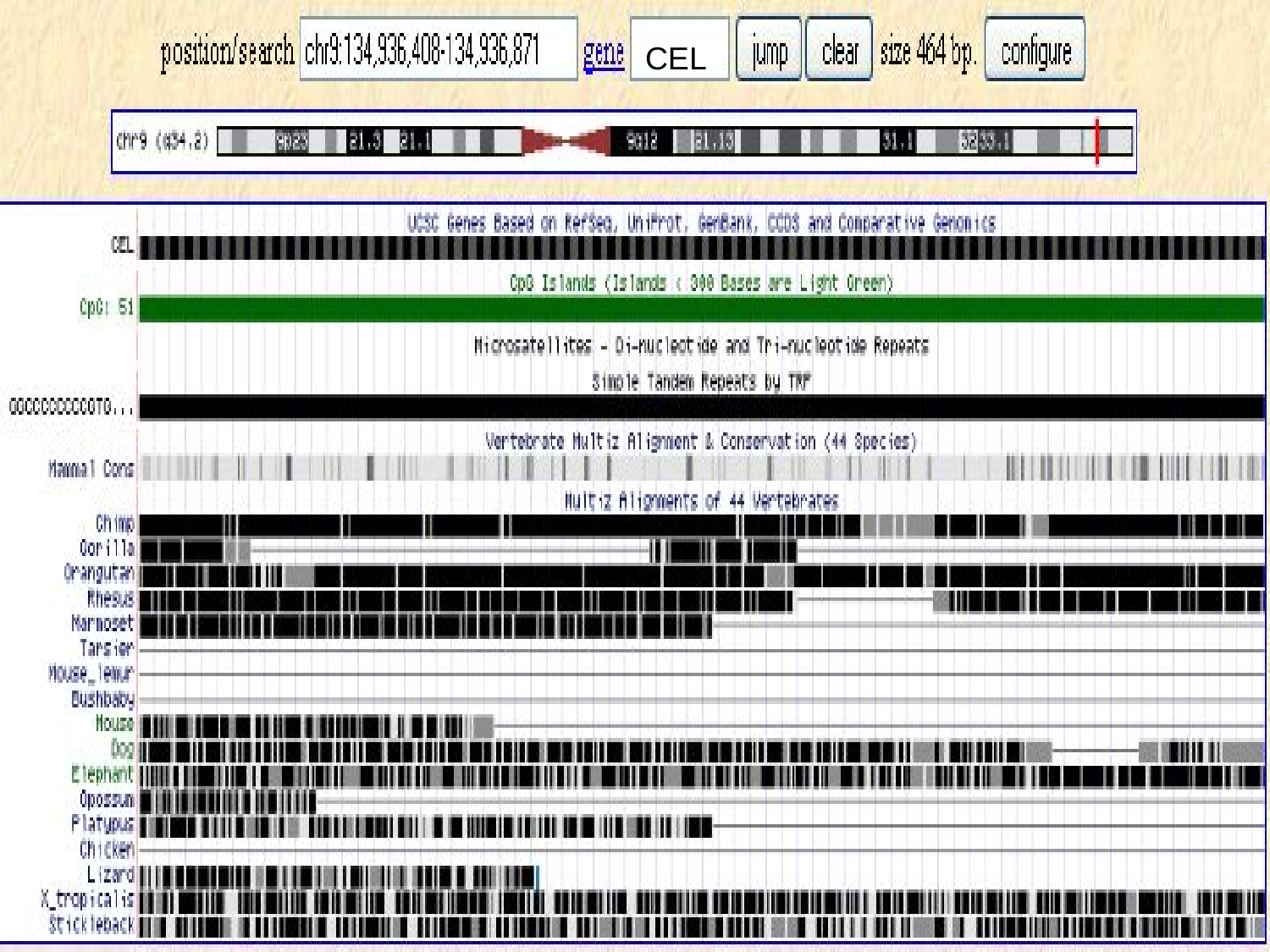

CEL

Supplement: Supplementary file 2 [file 781643.f2.ppt]
